# A Case of Rupture of a Muscular Attachment Followed by a Large Abscess

**Published:** 1885-04-20

**Authors:** W. A. Crow

**Affiliations:** Friendship, Va.


### A CASE OF RUPTURE OF A MUSCULAR ATTACHMENT FOLLOWED BY A LARGE ABSCESS.

By W. A. CROW, M.D., Friendship, Va.

A. B., male, age 35, laborer, six feet three inches high, and fine muscular development. Came to me on account of extreme soreness and pain on moving his right arm.

He gave the following history: About three weeks previous, while placing some billets of wood on his shoulder, he felt a sharp pain in his shoulder, running down to the lower end of shoulder blade; it lasted but a moment, so he thought nothing of it until next morning, when he found it gave him pain to move his shoulder, and that it was tender when he would press on it.

I found, on examination, tenderness near the head of humerus and over the inferior angle of scapula, together with some swelling and a little redness. The tender spots corresponded to the insertion of latissimus dorsi into the bicepital groove of humerus and inferior angle of scapula. I failed to detect either crepitus or false motion, so I diagnosed it rupture of the tendonous insertion of latissimus dorsi muscle into the bicepital groove of humerus.

While there was some puffiness over the inferior angle of the scapula, I could not detect fluctuation, but from the amount of pain and soreness, and from the time since he first noticed it, I thought it more than probable that pus would form, so I inserted my hypodermic needle full length, but failed to get anything but a drop of blood. I ordered him to have it painted three times a day with tincture of iodine until it got very tender. I also confined his arm at rest, gave some morphine to relieve the pain and calomel and ipecac to regulate bowels, and told him to see me again in four or five days.

A week passed before I again saw the case, when I found the swelling had increased considerably over the lower angle of scapula, and I could detect fluctuation. I drew off over a pint of pus with an aspirator, and washed out the cavity with a two-per-cent. solution of carbolized water (tepid), then placed a compress of cotton over the space by a roller bandage, still keeping his arm at rest; gave him tr. ferri chlor. gtt. x, tr. nucis vom gtt. 5, to be taken in a little water after eating, T. I. D.; also, to keep bowels regular and keep quiet for a week.

He made a good recovery and the pus never formed again.

